# Prey Preference of Snow Leopard (*Panthera uncia*) in South Gobi, Mongolia

**DOI:** 10.1371/journal.pone.0032104

**Published:** 2012-02-29

**Authors:** Wasim Shehzad, Thomas Michael McCarthy, Francois Pompanon, Lkhagvajav Purevjav, Eric Coissac, Tiayyba Riaz, Pierre Taberlet

**Affiliations:** 1 Laboratoire d'Ecologie Alpine, Centre National de la Recherche Scientifique, Unité Mixte de Recherche 5553, Université Joseph Fourier, Grenoble, France; 2 Snow Leopard Program, Panthera, New York, New York, United States of America; 3 Snow Leopard Conservation Fund, Ulaanbaatar, Mongolia; American Museum of Natural History, United States of America

## Abstract

Accurate information about the diet of large carnivores that are elusive and inhabit inaccessible terrain, is required to properly design conservation strategies. Predation on livestock and retaliatory killing of predators have become serious issues throughout the range of the snow leopard. Several feeding ecology studies of snow leopards have been conducted using classical approaches. These techniques have inherent limitations in their ability to properly identify both snow leopard feces and prey taxa. To examine the frequency of livestock prey and nearly-threatened argali in the diet of the snow leopard, we employed the recently developed DNA-based diet approach to study a snow leopard population located in the Tost Mountains, South Gobi, Mongolia. After DNA was extracted from the feces, a region of ∼100 bp long from mitochondrial 12S rRNA gene was amplified, making use of universal primers for vertebrates and a blocking oligonucleotide specific to snow leopard DNA. The amplicons were then sequenced using a next-generation sequencing platform. We observed a total of five different prey items from 81 fecal samples. Siberian ibex predominated the diet (in 70.4% of the feces), followed by domestic goat (17.3%) and argali sheep (8.6%). The major part of the diet was comprised of large ungulates (in 98.8% of the feces) including wild ungulates (79%) and domestic livestock (19.7%). The findings of the present study will help to understand the feeding ecology of the snow leopard, as well as to address the conservation and management issues pertaining to this wild cat.

## Introduction

Apex predators play a key role in maintaining biodiversity in an ecosystem, through population dynamics and trophic cascades [1], [2]. Despite a relatively wide distribution across 12 central Asian countries, information about snow leopards is scarce due to their remote habitat and cryptic nature [3]. The International Union for the Conservation of Nature (IUCN) Red List of Threatened Species has identified the snow leopard as being endangered since 1988; Appendix I of the Convention on International Trade in Endangered Species (CITES) has listed it as such since 1975. Its population is estimated to be between 4,500 and 7,500 throughout its range [4], with 800 to 1,700 in Mongolia [5]. Snow leopard populations are declining across most of its range. Primary threats include habitat degradation and fragmentation due to livestock grazing and human population expansion, poaching for pelts and bones, killings of snow leopards in retribution for predation on livestock, and reduction of natural prey populations due to illegal and legal hunting, as well as competition from livestock [6]–[8].

Diet analysis helps to reveal the plasticity of a predator's ability to both use and conserve resources available to it. This knowledge is important, especially when an animal is as endangered and secretive in nature as the snow leopard [9]. Snow leopards are considered to be opportunistic predators that exploit a wide range of prey species [10]. Large ungulates (notably blue sheep, markhor, urial, ibex, goats and sheep) often represent the major constituents of the snow leopard's diet. Additional prey items that have been observed include unidentified birds and a wide variety of medium and small mammals, such as marmots and other rodents [9], [11]–[14]. In Mongolia, Siberian ibex and argali are the natural prey of the snow leopard [15].

Detailed and accurate data on predators as well as their prey is required to assess the real magnitude of predation. This knowledge is imperative for the design of balanced conservation strategies.

To date, the diet of the snow leopard has been analyzed using different classical methods. Inference from field surveys, questionnaires and interviews with members of the local community can give an assessment of snow leopard predation and has been effectively used in some studies (e.g. [16], [17]). But such studies may only represent public opinion if they lack scientific confirmation. Radio telemetry provides a realistic opportunity to study animal movements, home range, pattern of habitat utilization, social organization [5] and to document predation by snow leopards [9], [18]. Although locating the remains of killed prey in high, steep terrain is extremely difficult [19], current GPS collaring studies of snow leopards in Mongolia (Panthera/Snow Leopard Trust unpublished data) have successfully identified kill sites and prey remains in over 250 instances between 2008 and 2011. However, that study is unique in its success and is not easily replicated across the snow leopard's broad range. Additionally, diet information from such studies remains site specific.

Fecal examination may then represent the most readily available and easily collected source of diet information [20]; this technique has been previously used to study snow leopard diet [7], [9], [11], [14]. Such diet analysis requires the identification of undigested remains, bones, teeth or hair in feces. There have been two potential problems relating to feces examination of the snow leopard. The first relates to the accurate identification of snow leopard feces in the field, while the second deals with the limitations of accurately identifying the prey taxa. Large bones and teeth are generally fragmented and therefore difficult to identify [21]. Hair examination is commonly done through comparisons of reference specimens with salvaged hair mounts [21]. However, this method is laborious and time consuming. Hairs from the same animal may also vary in structure according to their location within its fur. Similarly, hair from several related species may possess similar characteristics [21]. Finally, the lack of reference specimens can prohibit accurate diagnosis.

Snow leopard feces are identified in the field mostly on the basis of color, shape, location, pugmarks, scrapes, or the remains of prey species near the feces [11], [14]. However, carnivore feces are quite similar in their morphological characteristics; it is therefore not always easy to differentiate the feces among the sympatric carnivores [14], [22], [23]. To address this problem, some studies (e.g. [24], [25]) have used detection dogs trained to locate the feces of endangered species. In another study, scat detection dogs are being trained to distinguish snow leopard feces from other non-target feces after they have been collected in the field and shipped to the USA, thus eliminating the cost and complications of bringing a dog to the field. This may prove to be cost-effective for studies of snow leopards in which finding feces in the field is not difficult, but making a correct species identification is critical (McCarthy & Parker, unpublished data). But this approach is also prone to sample misidentification. Assumptions based on such errors may lead to incorrect conclusions regarding the conservation implications for these endangered species. Alternatively, feces collection in the field has been validated by genetic analysis [26], [27]. So far, two studies [7], [9] have identified snow leopard feces genetically and then determined prey consumed using classical approaches.

Livestock depredation has become a real challenge in central Asia throughout the range of the snow leopard [28]. Snow leopards are thought to be one of the major killers of livestock, which results in hostility towards the animal from local communities [16], [29], [30] and retribution killings of snow leopards [14], [31]. In a survey conducted in the four regions of Mongolia, 14% of livestock holders have admitted to hunting snow leopard to carry out retribution killings of their livestock [32]. Namgail et al. [17] have stated that 38% of the total livestock losses in Ladakh, India, can be attributed to snow leopards. Similarly, high proportions (40–58%) of livestock in snow leopard diet have been reported in two regions of India [14]. Due to these factors, local support of conservation efforts for this wild cat is questionable across its range.

This study aims to test two hypotheses about the snow leopard population from the Tost Mountains, South Gobi, Mongolia (i) Does livestock constitute the major part of the snow leopard diet? (ii) Does the snow leopard predate on threatened argali sheep? In our effort to address the potential limitations of the methods available to analyze the diet of snow leopards, we have benefited from the recent development of a DNA-based universal approach for diet analysis [33], which combines universal primers for vertebrates with a blocking oligonucleotide specific to snow leopard DNA and subsequent high-throughput next-generation sequencing. The results of the present study precisely identify all the prey items consumed, thus providing important knowledge for the conservation and management of this wild cat.

## Results

Of 203 putative snow leopard fecal samples collected in the field of Tost Mountains South Gobi Mongolia, 88 (43.3%) samples were identified as originating from this species using snow leopard specific primers (*UnciF/UnciR*); these were selected for further experimentation. After assembling the forward and reverse reads, and filtering for primers and tags, we obtained a total of 1900638 sequences for these 88 feces samples, corresponding to 173770 unique sequences (data deposited in the Dryad repository: doi:10.5061/dryad.bj376f61). Removing sequences shorter than 60 bp and with a total count inferior to 100 reduced the dataset to 463 sequences. Also removed were 364 sequences that were never “head” (most common sequence among all sequences that can be linked with a single indel or substitution) or “singleton” (no other variant with a single difference in the relevant PCR product), as were 74 sequences with a total count among all samples inferior to 1000 (these sequences only corresponded to variants of more common sequences). This threshold has been determined in order to avoid duplicative species identifications in the results list and with a lower identity for sequences below the threshold. Finally, 19 sequences were removed that lacked perfect identification from the reference database or were that of prey that had already been identified with higher count, leaving six (including one *Panthera uncia* and five prey sequences) MOTU (Molecular Operational Taxonomic Unit; [34]). Table 1 presents the overview of sequence counts at different stages of the analysis. The filtered data are available in the Dryad repository: doi:10.5061/dryad.bj376f61. All feces identified as snow leopard with the snow leopard specific primers (*UnciF/UnciR*) were further confirmed by sequencing; snow leopard sequences were observed in all of the samples with an overall relative frequency of 0.26. This means that the 2 µM concentration of blocking oligonucleotide used in this experiment reduced, but did not completely block the amplification of the snow leopard sequence (see Table 2 for a comparison of the results with and without blocking oligonucleotide). We observed seven samples that produced only the snow leopard DNA fragment but no prey count. Nine samples contained a sequence that corresponds to both argali and domestic sheep. These two species were prevalent in our study area. Further sequencing experiments with primer pair *OvisF/OvisR*, amplifying a 82 bp of the cytochrome *b* gene, had successfully identified argali and domestic sheep in seven and two samples, respectively.

**Table 1:** Overview of the sequence counts at different stages of the analysis.

	Number of reads (% of properly assembled sequences^a^)	Number of unique sequences^b^
Number of properly assembled sequences	1900638	173770
Filtering sequences length> = 60 bp & count> = 100	963714 (50.70%)	463
Filtering for most of the PCR/sequencing errors	841459 (42.27%)	74
Perfectly assigned taxa	725969 (38.19%)	6

a Direct and reverse sequence reads corresponding to a single DNA molecule were aligned and merged, producing what we called a “properly assembled sequence”.

b Strictly identical sequences correspond to “unique sequence”.

**Table 2:** Comparison of PCR amplification without and with blocking oligonucleotide.

	Snow leopard sequence (%)	Other sequences (%)
Experiments without blocking oligonucleotide	87.45	12.55
Experiments with blocking oligonucleotide	25.76	74.24

### Diet composition of snow leopard

The diet composition of snow leopards in the Tost Mountains of Mongolia was not highly diverse; a total of only five different prey items were identified in the diet. All prey taxa were identified to the species level. We observed one prey item per sample in all 81 samples, although we could not amplify any prey sequence in seven samples.

**Figure 1:**
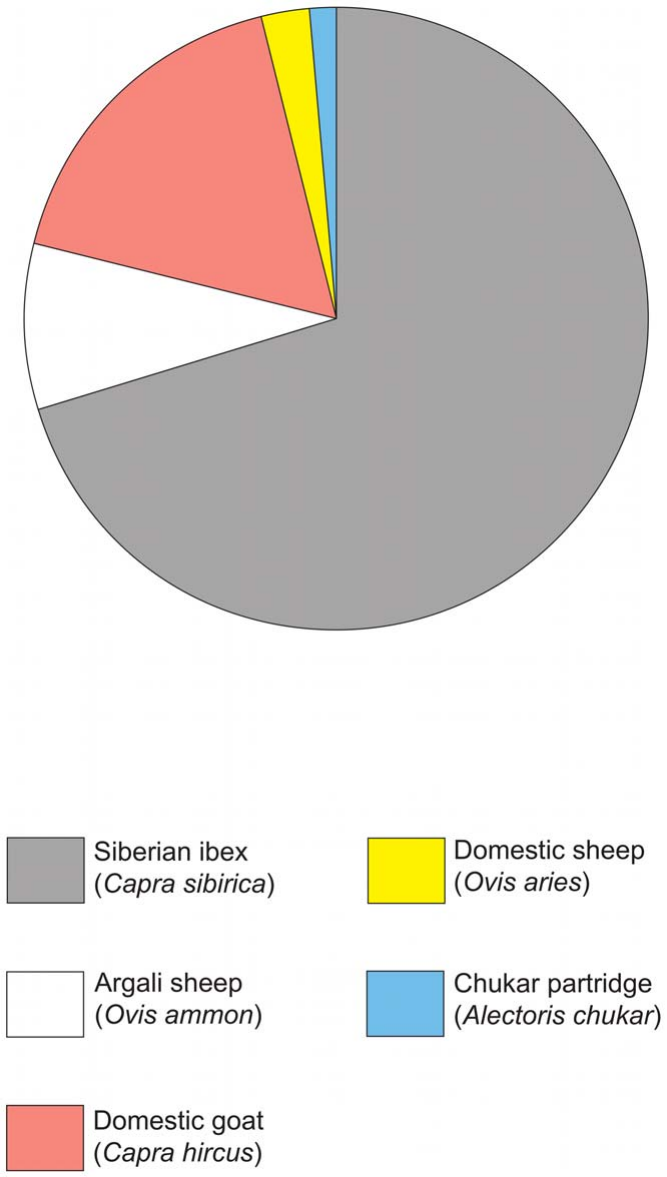
Relative frequencies of various prey species present in the diet, on the basis of their occurrence in feces of snow leopards from the Tost Mountains, South Gobi, Mongolia.

On the basis of its occurrence in feces, the Siberian ibex was observed to be the most frequent prey (70.4%), followed by domestic goat (17.3%) and argali sheep (8.6%). Overall, ungulates comprised the dominant part of the diet (in 98.8% of the feces) including wild species (79%) and domestic livestock (19.7%). Only one species of bird other than ungulates was recorded; a chukar partridge was recorded in one (1.2%) fecal sample (Figure 1). Table 3 presents an overview of the snow leopard diet in the Tost Mountains, South Gobi, Mongolia, in comparison with other studies.

**Table 3:** A comparison of frequency of occurrence (percent) of various prey items in the feces of snow leopard from various regions of its range.

Prey consumed	Present studySouth Gobi, Mongolia(n = 81)	Anwar et al. (2011)Baltistan, Pakistan(n = 49)	Bagchi & Mishra (2006) Pin Valley, India(n = 51)	Bagchi & Mishra (2006) Kibber, India(n = 44)	Lhagvasuren & Munkhtsog (2000) Uvs & South Gobi, Mongolia(n = 168)	Chundawat & Rawat (1994) Ladakh, India(n = 173)	Oli et al. (1993)Manang, Nepal(n = 213)
Wild ungulates							
Argali	8.6	-	-	-	-	-	-
Blue sheep	-	-	-	20.5	-	23.4	51.6
Goitered gazelle	-	-	-	-	3.6	-	-
Ladakh urial	-	-	-	-	-	0.4	-
Markhor	-	3.2	-	-	-	-	-
Red deer	-	-	-	-	2.4	-	-
Roe deer	-	-	-	-	0.6	-	-
Siberian ibex	70.4	9.7	56.9	9.1	38.7	-	-
Meso & small mammals							
Hare	-	-	3.9	6.8	1.2	3.1	-
Weasel	-	-	-	-	-	-	4.7
Marmots	-	-	-	-	1.2	9.8	20.7
Marten	-	-	-	-	-	-	3.8
Red fox	-	-	-	-	-	4.3	0.9
Pika	-	-	-	-	5.9	-	15.9
Rodents	-	-	-	-	0.6	-	-
Royale's vole	-	-	-	-	-	-	7.5
Domestic livestock							
Cattle & Yak	-	8.6	2.0	6.8	4.8	1.2	14.1
Donkey	-	-	3.9	13.6	-	0.4	-
Goat	17.3	11.8	3.9	9.0	3.6	10.2	0.5
Horse	-	-	11.8	4.5	5.4	0.8	2.8
Sheep	2.5	16.1	2.0	4.5	17.3	2.3	0.5
Birds	1.2	2.2	-	15.9	2.4	3.1	1.4
Insects	-	-	-	-	2.4	-	-
Plant matter	-	31.2	25.5	27.3	14.9	41.0	19.3
Unidentified matter	-	17.2	5.9	19.5	0.6	-	5.6

## Discussion

Our results are in general agreement with previous studies, indicating that large ungulates (wild and domestic) represent the major part of snow leopard diet (in 38.7–98.8%, of the feces). Moreover, Siberian ibex are most abundantly observed (9.1–70.4%) in the diet in many parts of snow leopard range except in the regions where ibex are rare [11], [12]. However, our results differ from other studies in that we are reporting high predation on wild ungulates (79%), which is considerably higher that previous reports (which range from 12.9–56.9%). It seems that snow leopard predation and plasticity depends upon the availability of its natural prey. Our study area appears to host an abundant population of wild ungulates, including Siberian ibex and argali sheep. It may be easier for snow leopard to attack wild prey than domestic, because the latter is often guarded by humans. In previously reported studies, medium and small mammals provide an important dietary supplement (3.9–53.3%). The absence of any medium or small mammals in our results cannot be considered to be an indication of preference, since we have no data on prey availability. Large wild cats tend to prefer large-sized prey [35]–[37]. Bird species represent an important element of snow leopard diet; although they are observed in low frequencies (1.2–15.9%), birds are consistent in all documented studies (Table 3).

Our findings support previous studies that snow leopard tend to focus primarily on a single prey item [11], [12]. We also observed only a single prey species per fecal sample. The average body weight of an adult snow leopard is about 45 kg [12], [38], for which the required daily prey biomass is estimated to be 1.5 to 2.5 kg [12]. Predation on large prey is therefore sufficient to fulfill its bodily requirements for several days. McCarthy [5] estimated that snow leopards kill a large prey item every 10–15 days and feed on it for an average of 3–4 days and sometimes up to one week.

Although this study only examined vertebrate prey items consumed by snow leopards, some studies [9], [11]–[14] have documented plant material in snow leopard feces (see also Table 3). Why snow leopards consume plant matter has not been definitively determined, although some studies [11], [13], [14] have suggested this phenomenon is the result of accidental ingestion while feeding on prey. The possibility has not been ruled out that plant matter fills some specific dietary need such as providing minerals or vitamins not readily gained from animal matter.

We observed seven samples that contained snow leopard DNA exclusively; no prey DNA could be amplified from them. A plausible explanation for this is found in that snow leopards may go several days between meals [5], [19] and in the latter part of this interval its feces would likely contain mostly hair (from grooming) and the cat's own metabolic waste products. Another difficulty arose in differentiating between argali and domestic sheep. These two species have the same sequence when using the universal primers for vertebrates *12SV5F/12V5R*. Both these prey species are found in the study area. To remedy this, we designed a new primer pair *OvisF/OvisR*, targeting a part of the cytochrome *b* gene from mitochondrial DNA. It showed consistent variation between these two potential prey species and helped to discern argali and domestic sheep remains.

DNA-based techniques provide a powerful means to study the feeding ecology of wild and cryptic species like the snow leopard. By using universal primers for vertebrates, limiting snow leopard sequences with a blocking oligonucleotide, and then using next generation sequencing, we were able to precisely identify all prey items to the species level. Traditional methods of snow leopard diet analyses via fecal samples have been unable to identify soft and well-digested components or the remains of specific bird species. Our approach has an obvious advantage in that virtually no vertebrate prey remained unidentified as the *12SV5F/12V5R* primers amplify 98% of all vertebrates [39].

### Conservation implications for snow leopards in Mongolia

A clear and unambiguous understanding of an endangered carnivore's diet is crucial for conservation planning for the species. To date, despite numerous studies, the diet of the snow leopard has been inadequately assessed. This has been true for at least two reasons: misidentifying feces as being those of snow leopards, and the inherent inaccuracies of classic macro- or micro-histological examination of fecal content. This study shows that both potential shortcomings can now be overcome through genetic techniques.

A better understanding of the diet of the snow leopard will allow us to more accurately assess the level of conflict between the cats and pastoralists who rightly or wrongly attribute their livestock depredation losses to snow leopards. Mitigating measures can then be designed that address a real, as opposed to a perceived, conflict.

In this study, argali, the largest wild mountain sheep, represented 8.6% of diet of snow leopards in the Tost Mountains. Argali are listed as “nearly threatened” in the IUCN Red List. Threats to the species, other then predation by snow leopards, include loss or degradation of habitat due to competition with domestic sheep and illegal hunting for meat and horns [40], [41]. In consideration of their prevalence in snow leopard diet, adequate conservation strategies are required to protect and increase the existing populations of argali in the Tost Mountains and elsewhere in the snow leopard range [42]–[44]. Illegal hunting should strictly be discouraged while habitat restoration for this wild sheep should be pursued.

The techniques we describe here can also be employed to help assess opportunities to increase snow leopard numbers in areas where they have been reduced. Knowledge of diet composition and prey availability in such instances would help conservationists determine if adequate wild prey is available to support hoped-for increases in snow leopard populations. This would help avoid situations where increasing leopard numbers only result in escalating conflicts with livestock and humans, dooming the effort to failure. Conversely, where conflict is already high and conservation efforts focus on reducing livestock depredation (predator-proof corrals, better guard dogs, etc.), an accurate assessment of current diet composition and wild prey availability would help avert unintended stress to snow leopards already facing inadequate food supplies to sustain their existing numbers.

## Materials and Methods

### Study area and sample collection

**Figure 2:**
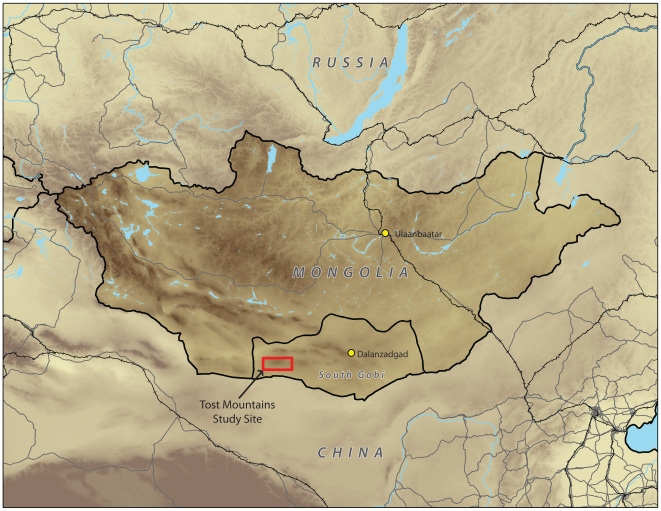
Location of Tost Mountain study site in South Gobi, Mongolia.

During the summer of 2009, 203 fecal samples were collected in the Tost Mountains of South Gobi province, Mongolia (Figure 2). The fecal collection, conducted by Panthera and the Snow Leopard Trust, was part of an ongoing long-term study of snow leopard ecology [45]. All necessary permits were obtained for the described field studies. Sampling authorizations in the South Gobi study area were provided by the Mongolian Ministry of Nature, Environment and Tourism. All samples were in 10 ml vials with ∼6 ml of silica gel. Each sample was later divided into three sub-samples for separate analysis. One sub-sample was submitted to the Global Felid Genetics Program at the American Museum of Natural History (AMNH) for species and individual identification through microsatellite analyses. A second sub-sample was sent to Working Dogs for Conservation (Bozeman, Montana, USA) to help train and test scat detection dogs in species identification. A third sub-sample was sent to the Laboratoire d'Ecologie Alpine (Université Joseph Fourier, Grenoble, France) for dietary analyses.

### DNA extraction

All extractions were performed in a room reserved for the extraction of degraded DNA. Total DNA was extracted from about 15 mg of feces with the DNeasy Blood and Tissue Kit (QIAgen GmbH, Hilden, Germany), following the manufacturer's instructions, with a slight modification at the beginning of the protocol as described by Shehzad et al. [33]. The DNA extracts were recovered in a total volume of 250 µL. Mock extractions without samples were systematically performed to monitor possible contaminations.

### Designing primer pairs for snow leopard diet study

#### Identification of predator species

A primer pair *UnciF/UnciR* highly specific to the snow leopard was designed on the 12S mitochondrial rRNA gene (Table 4), with the 3′-end of each primer as different as possible from other species. The specificity of this primer pair, amplifying a 68 bp fragment, was validated *in silico* by using the *ecoPCR* program [46], [47], with the following parameters: a perfect match on the two last nucleotides, and a maximum of three mismatches on the remaining nucleotides. Using these parameters, only *P. uncia* mitochondrial 12S gene was recovered. This *in silico* validation confirmed that snow leopard specific primers (*UnciF/UnciR*) should unambiguously identify snow leopard feces. The PCRs were carried out in a total volume of 20 µl with 8 mM Tris-HCl (PH 8.3), 40 mM KCl, 2 mM MgCl_2,_ 0.2 µM of each primer, BSA (5 µg), 0.5 U of AmpliTaq Gold® DNA polymerase (Applied Biosystems) using 2 µL as DNA template. The PCR conditions chosen were an initial 10 min denaturation step at 95°C, followed by 45 cycles of denaturation at 95°C for 30 s and annealing at 53°C for 30 s. Thus, the primary identification of the samples was done on the basis of the presence of a PCR product of the suitable length revealed by electrophoresis on a 2% agarose gel. The samples successfully amplified by the snow leopard specific primer pair (*UnciF/UnciR*) were selected for further analyses.

**Table 4:** Sequences of the primer pairs used in the study.

Name	Primer sequence (5′-3′)	Reference
*UnciF*	CTAAACCTAGATAGTTAGCT	Ficetola et al. 2010
*UnciR*	CTCCTCTAGAGGGGTG	Ficetola et al. 2010
*12SV5F*	TAGAACAGGCTCCTCTAG	Riaz et al. 2011
*12SV5R*	TTAGATACCCCACTATGC	Riaz et al. 2011
*UnciB*	CTATGCTTAGCCCTAAACCTAGATAGTTAG CTCAAACAAAACTAT-C3	This study
*OvisF*	AAACTATGGCTGAATTATCCGATA	This study
*OvisR*	TCCGATGTTTCATGTTTCTAGGAA	This study

The length of amplified fragments (excluding primers) with *Unci*, *12SV5* and *Ovis* were 68 bp, ∼100 bp and 82 bp, respectively.

#### Universal primer pair for vertebrates 12SV5F/12SV5R

We used the primer pair *12SV5F*/*12SV5R* (Table 4) designed by the *ecoPrimers* program [39]. The *ecoPrimers* scans whole genomes to find new barcode markers and their associated primers. This program optimizes two quality indices that measure the taxonomical coverage and the potential discrimination power in order to select the most efficient markers, according to specific experimental constraints such as marker length or targeted taxa. This universal primer pair for vertebrates represents the best choice found by *ecoPrimers* among short barcodes, according to the available vertebrate whole-mitochondrial genomes currently available. It amplifies a ∼100 bp fragment of the V5 loop of the mitochondrial 12S gene with the ability to amplify short DNA fragments such as recovered from feces, and which has a high taxonomic resolution despite its short size. Using the *ecoPCR* program [46], [47], and based on the 103 release of the EMBL database, this fragment unambiguously identifies 77% of the species and 89% of the genera.

#### Blocking oligonucleotide specific to snow leopard sequence

The *UnciB* (Table 4) blocking oligonucleotide sequence specific to snow leopards was designed as suggested by Vestheim & Jarman [48]. It overlaps the amplification primer *12SV5R* by six nucleotides. This blocking oligonucleotide was used to restrict the amplification of snow leopard sequences when using the universal primers that target all vertebrates.

#### Primer pair to distinguish Ovis aries and O. ammon

The two closely related prey species, domestic sheep (*Ovis aries*) and argali sheep (*O. ammon*), have potentially similar sequences for the amplified fragment of 12S ribosomal RNA gene. A special primer pair *OvisF/OvisR* (Table 4) targeting cytochrome *b* gene was designed to amplify a homologous mitochondrial DNA region (∼82 bp) that shows consistent variation between *Ovis aries* and *O. ammon*.

### DNA amplification for diet analysis

We performed experiments without and with the blocking oligonucleotide. All DNA amplifications were carried out in a final volume of 25 µL, using 2 µL of DNA extract as template. The amplification mixture contained 1 U of AmpliTaq Gold® DNA Polymerase (Applied Biosystems, Foster City, CA), 10 mM Tris-HCl, 50 mM KCl, 2 mM of MgCl_2_, 0.2 mM of each dNTP, 0.1 µM of each primer (*12SV5F*/*12SV5R*) and 2 µM for *UnciB* (*UnciB* only in the experiments with blocking oligonucleotide), and 5 µg of bovine serum albumin (BSA, Roche Diagnostic, Basel, Switzerland). The PCR mixture was denatured at 95°C for 10 min, followed by 45 cycles of 30 s at 95°C, and 30 s at 60°C; as the target sequences are ∼100 bp long, the elongation step was removed to reduce the +A artifact [49], [50] that might decrease the efficiency of the first step of the sequencing process (blunt-end ligation).

The universal primers for vertebrates *12SV5F* and *12SV5R* were modified by the addition of specific tags on the 5′ end to allow the assignment of sequence reads to the relevant sample [51]. All the PCR products were tagged identically on both ends. These tags were composed of CC on the 5′ end followed by seven variable nucleotides that were specific to each sample. The seven variable nucleotides were designed using the *oligoTag* program (www.prabi.grenoble.fr/trac/OBITools) with at least three differences among the tags, without homopolymers longer than two, and avoiding a C on the 5′ end in order to allow detection of possible deletions within the tag. All of the PCR products from the different samples were first purified using the MinElute PCR purification kit (QIAGEN GmbH). They were then titrated using capillary electrophoresis (QIAxel, QIAgen GmbH, Hilden, Germany) and finally, mixed together in equimolar concentration before the sequencing step.

### DNA sequencing

Sequencing was carried out on the Illumina Genome Analyzer IIx (Illumina Inc., San Diego, CA, 92121 USA), using the Paired-End Cluster Generation Kit V4 and the Sequencing Kit V4 (Illumina Inc.) and following manufacturer's instructions. A total of 108 nucleotides were sequenced on each extremity of the DNA fragments.

#### Sequence analysis and taxon assignation

The sequence reads were analyzed using the *OBITools* program (www.prabi.grenoble.fr/trac/OBITools). First, the direct and reverse reads corresponding to a single molecule were aligned and merged using the *solexaPairEnd* program, taking into account data quality during the alignment and the consensus computation. Then, primers and tags were identified using the *ngsfilter* program. Only sequences with perfect matches on tags and a maximum of two errors on primers were taken into account. The amplified regions, excluding primers and tags, were kept for further analysis. Strictly identical sequences were clustered together using the *obiuniq* program, keeping the information about their distribution among samples. Sequences shorter than 60 bp, or containing ambiguous nucleotides, or with a number of occurrences lower or equal to 100 were excluded using the *obigrep* program. The *obiclean* program was then implemented to detect amplification/sequencing errors, by giving each sequence within a PCR product the status of “head” (most common sequence among all sequences that can be linked with a single indel or substitution), “singleton” (no other variant with a single difference in the relevant PCR product), or “internal” (all other sequences not being “head” or “singleton”, i.e. corresponding to amplification/sequencing errors). Taxon assignation was achieved using the *ecoTag* program [52]. *EcoTag* relies on the FASTA35 program [53] to find highly similar sequences in the reference database. This database was built by extracting the relevant part of the mitochondrial 12S gene from the EMBL nucleotide library (release 107) using the *ecoPCR* program [47]. A unique taxon was assigned to each unique sequence. This unique taxon corresponds to the last common ancestor node in the NCBI taxonomic tree of all the taxids annotating the sequences of the reference database that matched against the query sequence. A final filtering was carried out by removing sequences that were never “head” or “singleton”, sequences that were not identified at the family level (for removing putative chimeras), and sequences with a total count among the whole dataset of less than 750 (plus the removing of an obvious human contamination). Finally, when a more precise identification was required, automatically assigned taxonomic identifications were completed manually by combining the automatic identification with distribution data of prey in the study area.

### DNA amplification and sequencing to differentiate between domestic and argali sheep

Fecal samples that amplified domestic or argali sheep sequences were re-amplified with primer pair *OvisF/OvisR* and re-sequenced, using capillary sequencing to distinguish between these two species.

PCR amplifications were conducted in a 20 µl volume with 2 mM MgCl2, 0.2 mM of each dNTP, 0.2 µM of each primer (*OvisF/OvisR*) and 0.6 unit of AmpliTaq Gold Polymerase (Applied Biosystems). After a 10 min period at 95°C for polymerase activation, 45 cycles were run with the following steps: 95°C: 30 s, 65°C: 30 s, without the elongation step. The PCR products were purified using the QIAquick PCR purification kit (Qiagen GmbH). Fifteen nanograms of purified DNA from this PCR product were used to sequence the *OvisF* and *OvisR* primers separately. Sequence reactions were performed for both DNA strands by using the ABI PRISM Dye Terminator Cycle Sequencing Reaction Kit (Applied Biosystems, Carlsbad, California 90028 USA) in a 20 µl volume with 0.2 µM of each primer. Twenty-five cycles were run with the following steps: 95°C: 30 s, 65°C: 30 s, without the elongation step. Excess dye terminators were removed by spin-column purification and the products underwent electrophoreses on an ABI 3I30xl PRISM DNA sequencer (Applied Biosystems) using the POP 7 polymer.
